# Modification of Anodic Titanium Oxide Bandgap Energy by Incorporation of Tungsten, Molybdenum, and Manganese In Situ during Anodization

**DOI:** 10.3390/ma16072707

**Published:** 2023-03-28

**Authors:** Marta Michalska-Domańska, Katarzyna Prabucka, Mateusz Czerwiński

**Affiliations:** Institute of Optoelectronics, Military University of Technology, 2 Kaliskiego Str., 00-908 Warsaw, Poland

**Keywords:** anodization, anodic titanium oxide, doping, incorporation of elements, bandgap energy, bandgap modification, photoactivity

## Abstract

In this research, we attempted to modify the bandgap of anodic titanium oxide by in situ incorporation of selected elements into the anodic titanium oxide during the titanium anodization process. The main aim of this research was to obtain photoactivity of anodic titanium oxide over a broader sunlight wavelength. The incorporation of the selected elements into the anodic titanium oxide was proved. It was shown that the bandgap values of anodic titanium oxides made at 60 V are in the visible region of sunlight. The smallest bandgap value was obtained for anodic titanium oxide modified by manganese, at 2.55 eV, which corresponds to a wavelength of 486.89 nm and blue color. Moreover, it was found that the pH of the electrolyte significantly affects the thickness of the anodic titanium oxide layer. The production of barrier oxides during the anodizing process with properties similar to coatings made by nitriding processes is reported for the first time.

## 1. Introduction

Anodization is an electrochemical process of metal surface oxidation, which is a relatively fast and inexpensive method for nanostructured oxide fabrication. During anodization processes, current flow is forced between the anode and cathode, and the anodic oxide grows on the metal surface (anode). The most studied materials produced by metal anodization are anodic aluminum oxide (AAO) and anodic titanium oxide (ATO), and in this study the focus is on ATO. Besides their chemical composition, the main differences between these two porous materials are their morphology and applications. While AAO typically consists of well-ordered and straight to the metal substrate nanocapillaries and is mainly used as a template for nanofabrication, ATO is composed of nanotubes and is used as a photocatalyst [1,2,3,4,5].

Generally, to prepare smooth and well-ordered anodic titanium oxide (ATO) in the form of nanotubes, a glycol-based electrolyte is applied with the addition of fluoride and water [6,7,8,9]. For titanium anodization, mainly third- and fourth-generation electrolytes are used to achieve this aim [10,11]. The best composition for these generations of electrolyte for titanium anodization is an NH_4_F concentration in the range of 0.1–1.0 wt%, addition of water at the level of 0.1–5 wt%, voltage in the range of 10–60 V, and room temperature [11]. Normally, the applied anodization conditions such as voltage, temperature, and time, as well as the experimental setup [12,13,14] and electrolyte age [15,16], affect the ATO morphology, structure, and properties [5,16,17,18,19,20,21,22]. For example, the change of electrolyte type to an alkaline one or change of the main solvent in the electrolyte from glycol to ethanol or glycerol [7,23] leads to the formation of anodic oxide film morphology with different characteristics to what is most known nowadays.

The scientific popularity of anodic titanium oxide (ATO) is due to its photoactive properties. The bandgap (BG) is the distance between the electron valence band and the conduction band and represents the minimum energy needed to excite an electron to a state in the conduction band where it can participate in conduction [24,25]. BG is a very important property of a material, because it gives it distinct properties determining whether it is an insulator, semiconductor, or conductor. In a semiconductor, BG determines its photoactivity, color, and conductivity [26]. Generally, titanium oxide is a semiconductor, and its bandgap depends on the crystallographic phase. Its bandgap is in the range of 3.02 eV to 3.20 eV [27,28,29], which corresponds to a narrow range of light wavelengths, from 385 nm (3.20 eV, anatase) to 405 nm (3.02 eV, rutile) [27]. This part of sunlight corresponds to the UV region, which covers only around 4% of the sun spectrum [30]. Changing a photocatalyst’s BG is realized to widen its application, and it can be carried out in various ways, mainly by semiconductor doping by a co-catalyst. For example, the doping of TiO_2_ photocatalysts by nanoparticulate co-catalysts (e.g., N, Fe, Cr_2_O_3_, Mn_3_O_4_, and Fe_2_O_3_) significantly improves the water splitting rate [24,25,29]. Doping of semiconductors can be realized by different methods, e.g., sol–gel, immersion techniques, or coprecipitation method [27,28,31,32,33]. For instance, it was reported that Rh-doped TiO_2_ synthesized via the immersion calcination process using rutile and anatase as a titanium substate [33] led to enhanced Rh-doped TiO_2_ activity in the hydrogen evolution reaction (HER) during electrochemical water splitting. The sol–gel method was applied to fabricate Ru- and Ta-doped TiO_2_, and it was shown that photocatalytic performance depends on the co-dopant ratio and that the average valence of dopants oscillates around +4 [33].

Although the methods mentioned above effectively increase the range of operation and effectiveness of TiO_2_-based photocatalysts, the search for simpler methods for photocatalyst modification is ongoing. One of the ideas is to carry out one-step or in situ modification of semiconductors during their fabrication.

In relation to this, an interesting phenomenon was observed during AAO preparation. It was reported that anodic aluminum oxide can be modified in situ during fabrication by incorporation of ions from the electrolyte into the oxide film [34,35,36,37,38,39,40,41]. It was observed that during anodization, the positively polarized anode attracted electrolyte species, mainly negatively charged ones. Then, these anions were trapped near the anode, and as a consequence, they were incorporated into the aluminum anodic oxide layer. In this manner, different elements (such as Cr or Cu) [37,38,42] as well as large particles such as indigo carmine have been incorporated into AAO [38].

In the case of titanium anodization, the elements can be incorporated into the anodic oxide from the electrolyte as well as from the substrate. For example, Wang et al. fabricated lanthanum/manganese-doped TiO_2_ in situ by one-step anodization and then used it as a photocatalyst in organic pollutant utilization with a degradation rate above 90% [43]. The La/Mn-doped ATO was fabricated from an electrolyte solution containing 50 mL glycerol, 50 mL H_2_O, 0.6077 g NH_4_F, 0.1244 g La(NO_3_)_3_, and 0.1244 g KMnO_4_, where La(NO_3_)_3_ and KMnO_4_ salts acted as lanthanum and manganese precursors, respectively. Mazierski et al. used an electrolyte composed of ethylene glycol (EG) with 2 vol% water, 0.09 M NH_4_F, and different weight percentages of urea (acting as a nitrogen precursor) to fabricate ATO doped by nitrogen [44]. Oxides prepared in that experiment were used as photocatalysts in the degradation of phenol. Although in the first cycle of photoactivity measurements ATO doped by nitrogen exhibited better activity than undoped oxide, with more reaction time the increased amount of carbon species blocked the surface of N-doped catalysts and as a consequence significantly reduced their photoactivity over time [44]. Shin et al. developed in situ fabrication of ATO doped by Ru for application as an electrode in electrolysis. They found that the highest amount of Ru was incorporated in the oxide at 60 V and that these samples demonstrated the highest electrocatalytic activity [45].

As it was mentioned before, it is possible to modify anodic titanium oxide during anodization by incorporation of alloying elements from the substrate [46,47]. It was found that oxide nanotubes made on a Ti-24Nb-4Zr-8Sn titanium alloy consisted of stoichiometric oxides of alloying elements: TiO_2_, Nb_2_O_5_, ZrO_2_, and SnO [47]. This observation was taken advantage of in another experiment, where a substrate of pure Ti was modified by rare-earth metals (RE metals = Ho, Er, Nd, Y, Ce, and Tm) to produce a Ti90RE10 alloy, and RE metals from the substrate were incorporated into ATO during Ti alloy anodization [48]. It was proven by EDS, XRD, XPS, and luminescence spectroscopy that in the described experiment well-ordered nanotubes made of a mixture of TiO_2_ and RE_2_O_3_ were fabricated during anodization. Moreover, it was shown that the as-prepared nanotubes after annealing at 450 °C exhibited photocatalytic and photoelectrochemical properties in phenol and toluene degradation [48]. Likewise, doping of anodic TiO_2_ realized during anodization by substrate elements has found applications not only in photocatalysis, but also in biomedicine. The composition of β-type biomedical titanium alloy substrates was modified by other elements (such as Ti-30Ta [49], Ti–7.5Mo [50], Ti-15Mo [51], and Ti-35Nb and Ti-35Nb-4Sn [52]) with subsequent anodization to improve the biological response of those materials. In all the abovementioned studies [49,50,51,52], nanotubular structures grew on top of the alloy, and its biomedical performance was improved in comparison to un-anodized samples. Unfortunately, analysis of ATO composition was beyond the scope of those investigations. Another study reported the incorporation of alloying elements from biomedical Ti alloys (namely Ti6Al4V and Ti6Al7Nb) into ATO fabricated on top of the alloy [34].

In the presented research, we attempted to modify the bandgap of anodic titanium oxide by in situ incorporation of selected elements into the anodic oxide during the titanium anodization process. The proposed method of ATO modification seems to be efficient and easier than procedures used to tune the bandgaps of semiconductors mentioned in available reports. The purpose of this study is to investigate the change in the bandgap of anodic titanium oxide realized by modifying ATO in situ with selected elements. The goal is to obtain a bandgap of ATO with promising photoactivity over a wider wavelength range of sunlight than UV.

## 2. Materials and Methods

The titanium foil with technical purity (0.25 µm, 99.5%, Alfa Aesar, Haverhill, MA, USA) was cut into a rectangle with dimensions of 16 × 20 mm and was used as a substrate in anodization processes. A platinum grid was used as a cathode, and the titanium rectangle was used as an anode. Before anodization, titanium substrates were cleaned in a mixture of ethanol and isopropanol and then air-dried. One-step anodization was conducted in an electrochemical cell connected to a thermostat (HUBER, model MPC-K6) to provide constant temperature measurement during titanium anodization. A multimeter (RIGOL DM3058E, Batronix, Preetz, Germany) was used to measure and store the registered current data. The anodization electrolyte, named the reference electrolyte, contains 0.1 M of ammonium fluoride NH_4_F (>98%, Sigma-Aldrich, St. Louis, MI, USA), 2 vol% of deionized water (DI), and ethylene glycol as a solvent. To analyze the possibilities for in situ ATO modification during fabrication, the following pure salts were used as electrolyte dopants: Mn(NO₃)₂ (>97%, Chempur, Piekary Śląskie, Poland), Na₂MoO₄ (>98%, Sigma-Aldrich), Na₂WO₄ (>99%, Sigma-Aldrich). The concentration of dopants was kept at the same level in all electrolytes and was established at 0.01 M. To allow incorporation of manganese (Mn) from Mn(NO₃)₂ into ATO, the charge of the manganese ions (Mn^2+^) has to be changed to negative. To achieve this, it was decided to transfer manganese cations to the negatively charged complex. For this purpose, the addition of 0.02 M of tartaric acid (C_4_H_6_O_6_, 99.5%, Sigma-Aldrich), which acts as a complexing agent, was applied. Table 1 presents the composition of all electrolytes used in these experiments.

The pH and conductivity of each electrolyte solution were measured 3 times by a pH and conductivity meter (Metler Toledo, model Seven Compact Duo). The anodic oxide films were prepared at various voltages (20 V, 40 V, 60 V), at a constant temperature of 10 °C, and for a constant time of 1 h. To control the applied anodization voltage, adjustable DC power supply (Delta Elektronica, Zierikzee, The Netherlands, model MS1500 MS 400-AR-R, Poland) was used. During each anodizing process, magnetic stirring of the electrolyte with a speed of 200 rpm was used.

The presented oxide thickness is an average taken from 15 measurements made by a coating thickness gauge with Iso Scope probes (Fisher, Schwerte, Germany). Characterization of the anodic TiO_2_ morphology and of its chemical composition was executed using a scanning electron microscope, Quanta 3DFEG (FEI, Hillsboro, OR, USA), equipped with an energy-dispersive X-ray spectroscopy (EDS) detector. The presented chemical composition is an average taken from three measurements collected from various places on samples, each from areas of 600 × 600 µm^2^, at a working distance of 12 mm, beam energy of 30 kV, and magnification of 500×. The average nanopore diameter was estimated using ImageJ software (ver.1.53a, Madison, WI, USA). A UV-Vis spectrometer (Perkin Elmer, Waltham, MA, USA, model Lambda 650) was used to measure diffuse reflectance, and Tauc plots were used to estimate the bandgap of anodic oxides prepared in this study. More information about Tauc plots is given in the Section 3. The UV-Vis measurements were made in the wavelength range of 200–800 nm, at room temperature, and with a measuring step of 0.5 nm.

## 3. Results and Discussion

### 3.1. Characterization of Electrolytes

Before being used for titanium anodization, the electrolytes used in this study were characterized. Since Table 1 presents the detailed composition of the used electrolytes, in Table 2 the pH and the conductivity of tested electrolytes are presented. pH varied from slightly acidic for electrolyte A (with addition of Mn(NO_3_)_2_), to almost neutral for the reference electrolyte, to alkaline for electrolytes C (with addition of Na₂WO₄) and B (with addition of Na₂MoO₄). Conductivity is treated as the ability of a material to conduct an electric current and depends on the available charge carriers (ions) in electrolytes and their mobility, the valence of available ions, and the temperature of electrolytes [53]. In the present research, the temperature of electrolytes was kept constant in all tests (10 °C), and differences in conductivity are the result of the ions’ mobility and the valency of ions. It was observed that the conductivity of the electrolyte increases in the following order: A < B < Reference < C, which is not in line with the growth trend of pH.

### 3.2. Characterization of Anodic Oxides

**Figure 1:**
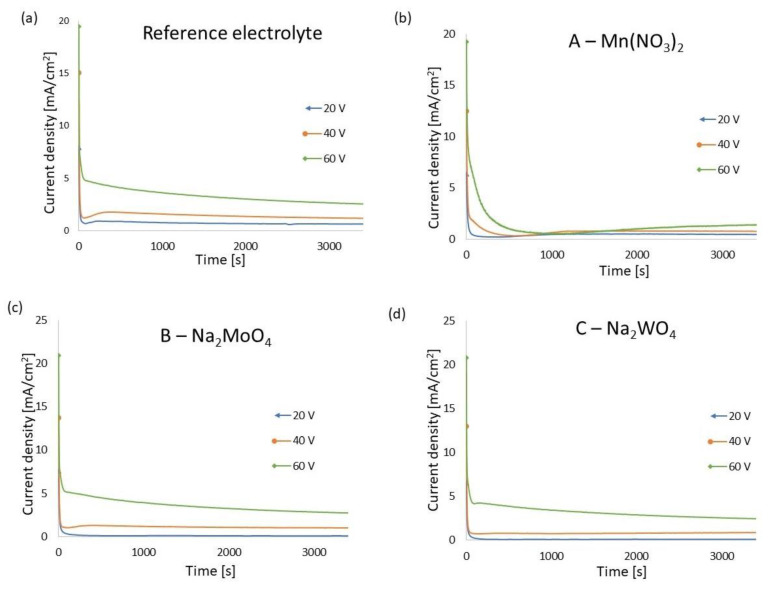
Current curves registered during anodization of titanium in reference (**a**), A—Mn(NO_3_)_2_ (**b**), B—Na₂MoO₄ (**c**), and C—Na₂WO₄ (**d**) electrolytes at 20 V, 40 V, and 60 V.

During anodization, the changes in current density as a function of anodization time, named current curves, were recorded, and are presented in Figure 1. The course of the current curve in all experiments is typical for titanium anodization. Generally, it consists of the following stages: at the beginning of anodization, on the anode surface the dense barrier type of anodic oxide grows and current density increases to reach the maximum (stage I). Next, in the oxide layer irregular nanopores begin to form and grow, and current density decreases (stage II). Subsequently, regular nanotubes are initiated and current density grows slightly to reach a steady stage (stage III) [7,54]. In previous research, a stage IV on current curves registered for aluminum anodization was postulated, which was associated with increased incorporation of ions into anodic oxide [55]. In this research, stage IV is visible on current curves registered for anodization in electrolyte A—Mn(NO_3_)_2_ with applied voltages of 20 V and 40 V (compare Figure 1a).

In Table 3, the average current density (mA/cm^2^) and the maximum current density (mA/cm^2^), depending on the applied electrolyte type and voltage, are presented. It can be noticed that in every tested electrolyte, both the average and maximum current density increased with the increase in applied voltage. The lowest value of average current density was registered for electrolytes B—Na₂MoO₄ and C—Na₂WO₄ at a voltage of 20 V. Additionally, for applied voltages of 40 V and 60 V and for all tested electrolytes, the significantly smallest values of average current density were registered for samples made in electrolyte A—Mn(NO_3_)_2_. Interestingly, the pH as well as conductivity of electrolyte A—Mn(NO_3_)_2_ was also the lowest (compare Table 2). Apart from the 20 V voltage and samples from electrolyte A—Mn(NO_3_)_2_, the average current density for respective voltages recorded for the other tested electrolytes was at a similar level.

**Figure 2:**
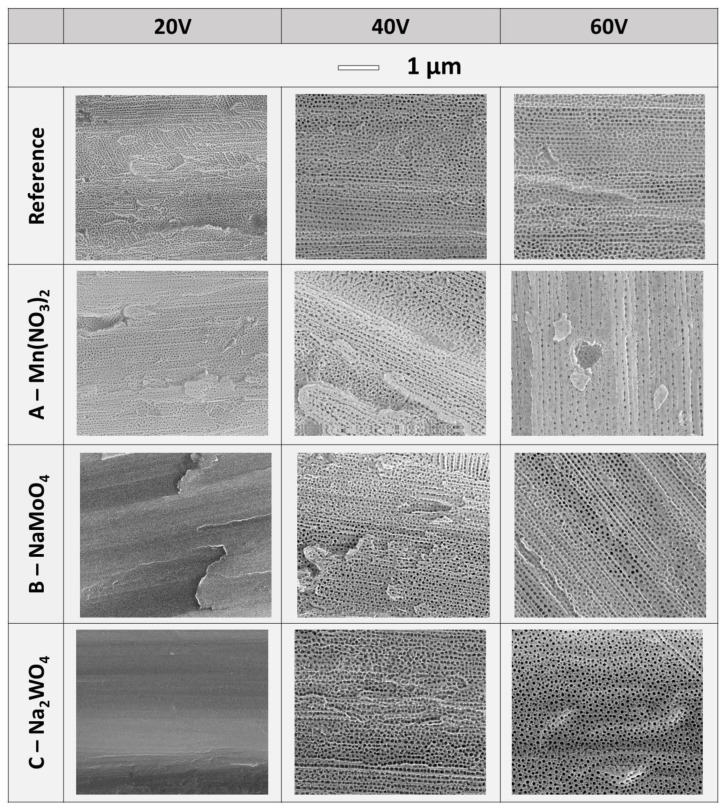
Morphology of anodic titanium oxide depending on the used electrolyte and the applied voltage.

The morphology of obtained anodic titanium oxides is presented in SEM images shown in Figure 2. It was proved that the main morphology of anodic titanium oxide obtained in all tested electrolytes is poorly arranged nanotubes. The exceptions are the anodic oxides produced in electrolytes B—Na₂MoO₄ and C—Na₂WO₄ at applied voltage of 20 V. In those cases, only barrier-type anodic oxides, without nanotubes or nanopores, were grown. Interestingly, the average current density recorded during the production of those barrier-type oxides was the lowest from all experiments (compare Table 3, 0.12 mA/cm^2^ for electrolyte B—Na₂MoO₄ and 0.13 mA/cm^2^ for electrolyte C—Na₂WO₄). The next value of average current density was almost 4 times higher than registered for barrier-type oxides, and it was 0.44 mA/cm^2^ for electrolyte A—Mn(NO_3_)_2_ for applied voltage of 20 V. On the surface of samples anodized in electrolyte A—Mn(NO_3_)_2_ at 20 V, nanotubes were observed.

**Figure 3:**
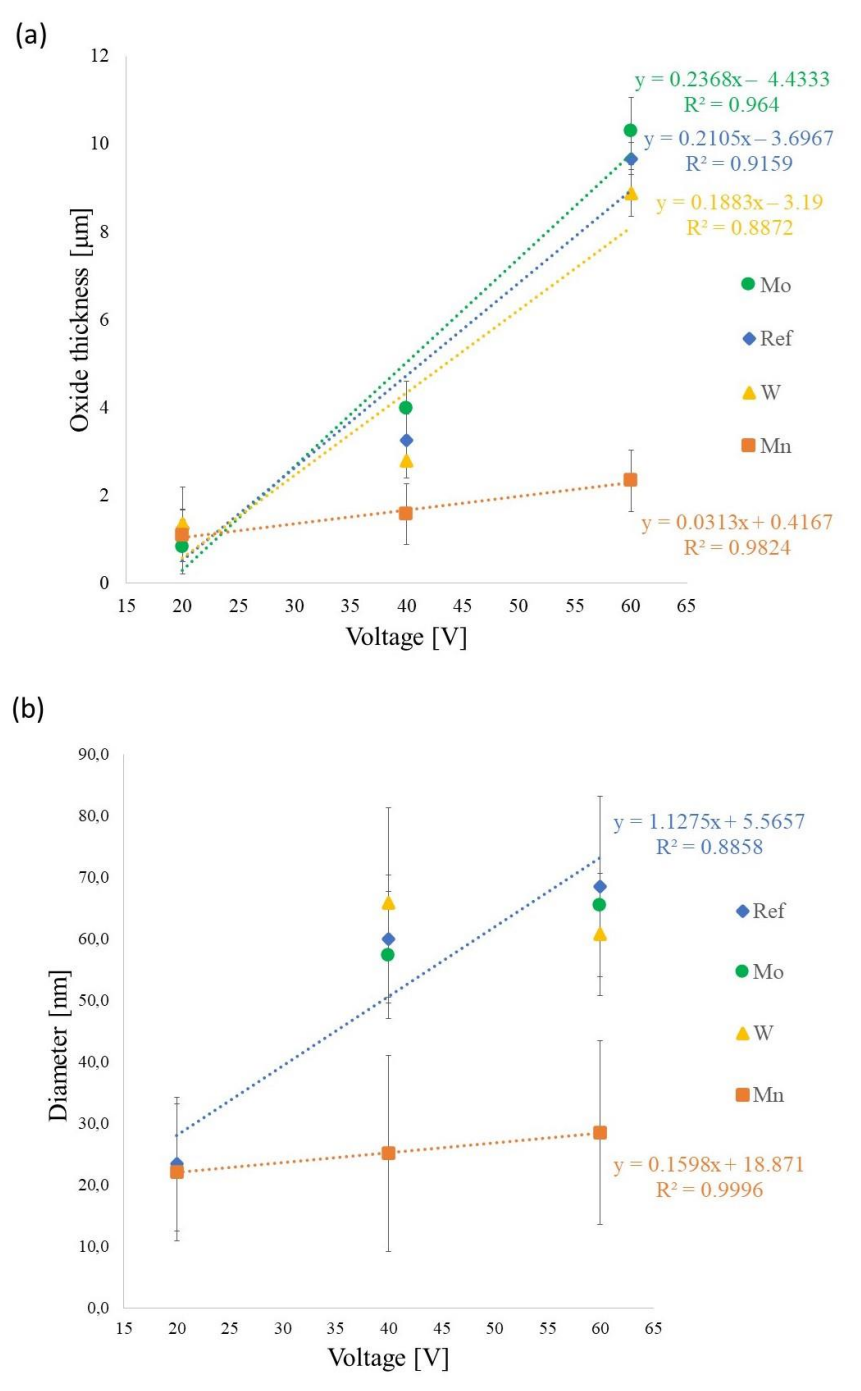
Thickness of anodic titanium oxide (**a**) and nanotube diameter (**b**) depending on the applied voltage and electrolyte.

The anodic titanium oxide thickness and the nanotube diameter were measured, and the results are presented in Figure 3. As is usually the case for materials fabricated by anodization, the thickness of anodic oxides increases with applied voltage (Figure 3a). Generally, the samples made in electrolytes B—Na₂MoO₄ and A—Mn(NO_3_)_2_ have the greatest and the smallest thickness, respectively. The tendency of thickness change is similar for samples prepared in the reference electrolyte and electrolytes with molybdenum (B—Na₂MoO₄) and tungsten (C—Na₂WO₄).

A significantly lower curve slope of thickness change was observed for samples fabricated in electrolyte A—Mn(NO_3_)_2_. It is worth noting that the pH of electrolyte A—Mn(NO_3_)_2_ was the lowest (5.35) and that this solution was acidic in nature. In such an environment, the oxide layer was probably being dissolved faster than it was growing, which affected the final oxide thickness. Except for the voltage of 20 V, the average current density registered in electrolyte A—Mn(NO_3_)_2_ was the smallest, which could have impacted nanotube diameter in the anodic layer. As it is shown in Figure 3b, the nanotube diameter increases with applied voltage for samples fabricated in reference and A—Mn(NO_3_)_2_ electrolytes. Because in electrolytes B—Na₂MoO₄ and C—Na₂WO₄ at 20 V only the barrier type of oxide was grown, and only two values of diameter were estimated (for 40 V and 60 V), the curve of diameter change depending on applied voltage is not plotted. However, it can be noticed that the nanotube diameter estimated for samples prepared in electrolyte B—Na₂MoO₄ tends to increase with increasing voltage. In the case of anodic films prepared in electrolyte C—Na₂WO₄, the nanotube diameter was 65.9 ± 15.4 nm and 60.7 ± 9.9 nm for 40 V and 60 V, respectively. The small difference in the nanotube diameter size combined with the relatively large standard deviation puts both values within their error limits. Therefore, under these specific anodization conditions, trends in diameter changes cannot be inferred.

To confirm incorporation of Mn, Mo, and W into the anodic titanium oxide layer, analysis of the chemical composition of samples was performed by EDS. The average chemical composition of fabricated oxide is given in Table 4. It was found that samples produced in modified electrolytes (A—Mn(NO_3_)_2_, B—Na₂MoO₄, and C—Na₂WO₄) contained the selected element (Mn, Mo, or W) in their chemical composition. Generally, in all experiments the content of titanium decreases while the contents of carbon and fluoride gradually increase with applied voltage. It must be noted that depending on the material density and beam voltage (beam energy), the practical electron range (penetration depth) and the X-ray range during EDS measurements are different [56]. Based on the Anderson–Hasler equation [57], in the case of titanium oxide, the penetration depth at beam energy of 30 kV is 4.17 µm. In this study, the thickness of anodic oxide depends on the applied voltage, and it was in the range of 0.83–10.30 µm (compare Figure 3). This means that during EDS measurements, in experiments made at 40 V almost all signal and at 60 V all signal comes from the anodic oxide layer. Therefore, the decrease in titanium content with the increase in applied voltage is related to anodic oxide thickness growth: The higher the oxide thickness, the smaller the impact of the Ti substrate on the whole amount of Ti in EDS measurements. On the other hand, the increases in carbon and fluoride contents with the increase in applied voltage are related to the growth of oxide thickness as well as higher incorporation of electrolyte species into the anodic oxide with the increase in applied voltage. The tendency of the oxygen amount to increase with the increase in applied voltage is consistent with the change in the contents of C and F, with a slight fluctuation observed for samples prepared in electrolyte A—Mn(NO_3_)_2_.

Some interesting observations were made for the content of nitrogen in tested oxides. Generally, the source of the nitrogen in fabricated ATO is NH_4_F in all electrolytes and additionally Mn(NO_3_)_2_ in electrolyte A. Except for barrier-type oxides made at 20 V in electrolytes B—Na₂MoO₄ and C—Na₂WO₄, the content of nitrogen in studied samples increases with applied voltage, which is typical for anodization processes. However, for those barrier-type oxides, the content of nitrogen was extremely high, and simultaneously the carbon and oxygen contents were surprisingly low, as well as the amount of fluorine being significantly reduced compared with the rest of the measurements (compare highlighted part of Table 4). Probably, in those specific anodization conditions (electrolytes B—Na₂MoO₄ and C—Na₂WO₄ at 20 V and 20 °C), electrochemical oxidation was not the preferred reaction. Perhaps nitriding was preferred for thermodynamic reasons. Normally, coatings similar to the barrier-type oxides obtained in this study are known as nitriding layers [58,59]. Basically, nitriding is a surface thermochemical treatment in which nitrogen is diffused into the surface layer of a solid alloy. The aim of nitriding is to improve the superficial mechanical and anticorrosion properties of materials and still maintain the bulk properties (such as mechanical strength, density, impact resistance, etc.). There are many methods for carrying out nitriding processes, which usually require high temperature and/or pressure in the atmosphere of a nitrogen source, such as N_2_ or NH_3_ plasma treatment or the rapid thermal process (RTP) in a nitrogen gas environment [58]. It is worth noting that Baker mentioned previously [60] that nitridation can give concurrent results with oxidation in the air, or in combustion environments employing air as the oxidizing gas in the case of steel, especially when steel has additional elements such as aluminum, titanium, and niobium. In that case, the nitrides formed are typically internal, which is particularly favorable thermodynamically [60]. Nevertheless, this occurred in the gaseous oxidizing of steel, not in the electrochemical oxidation of titanium. It should be remembered that in the present study, the reaction temperature was low (20 °C) and the environment was liquid, consisting mainly of ethylene glycol, water, and small additions of salts (NH_4_F, Na_2_MoO_4_, or Na_2_WO_4_), as well as the anodization conditions being gentle, giving a very low current density and did not generate plasma on the anodized samples. In summary, on the basis of the presented results and the best knowledge of the authors, it can be concluded that during Ti anodization under the specific anodization conditions, a nitrogen-rich coating was fabricated, which is reported for the first time. More tests should be conducted in future to explain this phenomenon in detail; however, this is beyond the scope of this paper.

### 3.3. Bandgap Investigation

As mentioned above, depending on the crystalline phase, the bandgap (BG) of TiO_2_ ranges from 3.0 to 3.2 eV, and for nanomorphological samples it can even be shifted to a higher value [29]. The given BG range corresponds to UV light, which covers only approximately 4% of all sunlight [30]. One of the commonly used methods for widening BG range is doping of photocatalysts by co-catalysts.

**Figure 4:**
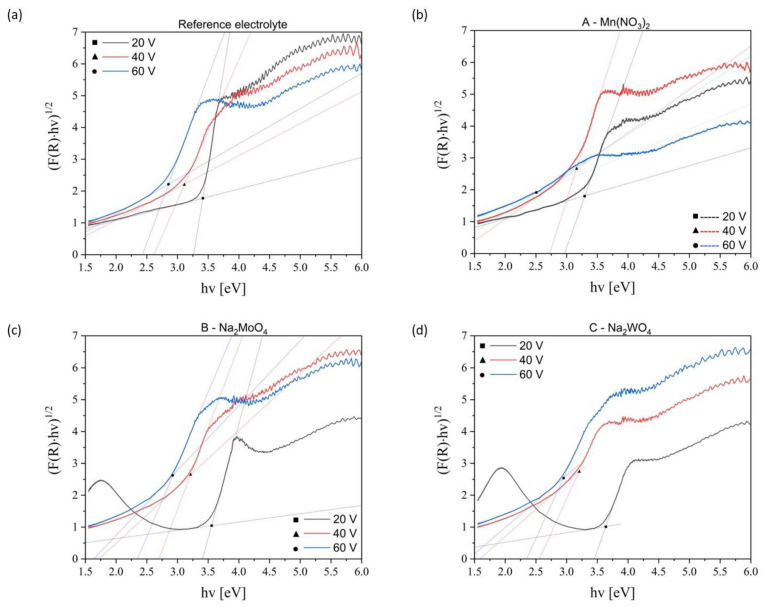
The Tauc plots used to estimate the bandgap of prepared anodic titanium oxide prepared in reference (**a**), A—Mn(NO_3_)_2_ (**b**), B—Na₂MoO₄ (**c**), and C—Na₂WO₄ (**d**) electrolytes at 20 V, 40 V, and 60 V.

In the present research, it was proved that the selected elements were incorporated into ATO, which should lead to a change in BG in the resulting samples. The Tauc plot was used to determine the bandgap of the prepared doped ATO. The curves used for this evaluation are presented in Figure 4. The x-axis shows the energy—hv in electron volts, while the y-axis shows the Kubelka–Munk function calculated from the measured diffuse reflectance of the ATO sample. By approximation with a linear function, a particular section of the graph, and using an additional levelling line, the value of estimated bandgap of the doped ATO was determined [61]. It was shown that the obtained BG value varied depending on the sample preparation conditions. In order to facilitate BG change analysis, the obtained results are summarized in Table 5.

It is worth noting that in all tested electrolyte compositions, the estimated BG of the anodic oxide is inversely proportional to the voltage applied during its preparation. It can be stated as a rule that the higher the voltage, the lower the BG. Moreover, the following trend can be declared: the BG level is similar in samples made under the corresponding conditions in all electrolytes. For example, the BG of samples made at 40 V is 3.14, 3.16, 3.21, and 3.15 eV for reference, A—Mn(NO_3_)_2_, B—Na₂MoO₄, and C—Na₂WO₄ electrolytes, respectively (compare Table 5). The BG values for samples prepared at voltages of 20 V and 40 V for all tested electrolytes are in the UV region of sunlight. The highest BG values were found for the barrier type of anodic oxide, made in electrolytes B—Na₂MoO₄ and C—Na₂WO₄ at an applied voltage of 20 V. The observed behavior of the barrier type of oxide is probably related to its unusual composition, which will be the subject of future research. In contrast, anodic oxides fabricated at 60 V in all tested electrolytes have a BG in the Vis region of sunlight. Among them, a sample made in electrolyte containing manganese stands out. This modified anodic titanium oxide had the smallest BG value of 2.55 eV. What is important is that compared with the rest of the doped and reference samples, the bandgap of the anodic titanium oxide with Mn prepared at 60 V is the most significantly shifted to the visible sunlight region. The recorded value of BG (2.55 eV) corresponds to a wavelength of 486.89 nm and blue color. The conditions for the fabrication of this oxide are as follows: 60 V in electrolyte A—Mn(NO_3_)_2_, which has the lowest pH (5.35) and conductivity (408.5 µS/cm) of all tested electrolytes; lowest average current density registered for 60 V from all tested electrolytes; the Mn content in the formed anodic oxide was at the level of 0.13 at % (compare Table 2, Table 3 and Table 4).

## 4. Conclusions

In this research, we attempted to modify the BG of anodic titanium oxide by incorporation of selected elements into the anodic titanium oxide in situ during the anodization process. Based on the obtained results, the following conclusions can be stated:

(1) Incorporation of Mn, Mo, and W into the anodic titanium oxide in situ during anodization was confirmed.
(2) The incorporation of the selected elements changes the bandgap of the anodic titanium oxide. The BG level was similar between samples made in corresponding conditions in all electrolytes.
(3) For all tested dopants, the BG values of samples prepared at voltages of 20 V and 40 V are in the UV region, while BG values of oxides made at 60 V are in the Vis region of sunlight.
(4) The smallest bandgap value was obtained for anodic titanium oxide made at 60 V modified by manganese (Mn), at 2.55 eV, which corresponds to a wavelength of 486.89 nm from the Vis region of sunlight (blue color).
(5) The pH of the electrolyte solution significantly affects the growth of the anodic titanium oxide layer. In an electrolyte with neutral pH, it is possible to obtain the thickest oxide.
(6) For the first time, barrier-type oxides made on a Ti surface at 20 V in electrolytes B—Na₂MoO₄ and C—Na₂WO₄, showing an extremely high content of nitrogen, are reported. The observed compositions of the oxides are similar to the compositions of coatings made in nitriding processes, but the anodization conditions during their preparation are significantly more gentle than during nitriding.

## Figures and Tables

**Table 1:** Composition of tested electrolytes.

Electrolyte Name	Base	Concentration			
		0.1 M	0.01 M	0.02 M	2 vol%
Reference electrolyte	Ethylene glycol	NH_4_F	-	-	H_2_O
A—Mn(NO_3_)_2_			Mn(NO_3_)_2_	C_4_H_6_O_6_	
B—NaMoO_4_			Na_2_MoO_4_	-	
C—Na_2_WO_4_			Na_2_WO_3_		

**Table 2:** pH and conductivity of tested electrolytes.

Electrolyte Name	pH	Conductivity (μS/cm)
A—Mn(NO_3_)_2_	5.35	408.5
Reference	6.61	607.6
C—Na_2_WO_4_	7.59	622.7
B—Na_2_MoO_4_	7.84	599.5

**Table 3:** The average current density (mA/cm^2^) and the maximum current density (mA/cm^2^) depending on the applied electrolyte and voltage.

Electrolyte Name	Applied Voltage (V)	Average Current Density (mA/cm^2^)	Maximum Current Density (mA/cm^2^)
Reference	20	0.73	8.20
	40	1.44	15.06
	60	3.31	19.46
A—Mn(NO_3_)_2_	20	0.44	6.61
	40	0.76	12.46
	60	1.31	19.23
B—Na_2_MoO_4_	20	**0.13**	**7.91**
	40	1.12	13.73
	60	3.57	20.92
C—Na_2_WO_4_	20	**0.12**	**6.85**
	40	0.82	12.97
	60	3.14	20.78

**Table 4:** The average element content (at %) in the ATO fabricated in different electrolytes as a function of the applied anodization voltage, measured by EDS.

Electrolyte Name		Element (at %)	Ti	C	N	O	F	Mn	Mo	W
	Voltage (V)									
Reference electrolyte	20 V		37.40 ± 0.15	12.62 ± 0.32	13.52 ± 1.42	30.67 ± 0.67	5.80 ± 0.13	-	-	-
	40 V		32.58 ± 0.18	13.61 ± 0.14	11.70 ± 1.05	35.68 ± 0.84	6.44 ± 0.17	-	-	-
	60 V		24.64 ± 0.47	14.06 ± 0.52	12.69 ± 0.57	40.44 ± 0.38	8.19 ± 1.18	-	-	-
A—Mn(NO_3_)_2_	20 V		36.76 ± 0.46	10.06 ± 0.49	11.40 ± 2.50	36.91 ± 2.36	4.65 ± 1.05	0.23 ± 0.04	-	-
	40 V		35.29 ± 073	10.36 ± 0.43	12.48 ± 0.64	35.44 ± 1.10	6.27 ± 0.69	0.17 ± 0.01	-	-
	60 V		31.95 ± 0.18	10.92 ± 1.15	12.83 ± 0.23	38.52 ± 0.92	5.65 ± 0.28	0.13 ± 0.01	-	-
B—Na_2_MoO_4_	**20 V**		**48.55 ± 3.78**	**9.70 ± 1.12**	**26.35 ± 2.56**	**12.88 ± 6.77**	**2.24 ± 0.72**	**-**	**0.29 ± 0.03**	**-**
	40 V		33.04 ± 0.55	12.86 ± 1.12	12.77 ± 2.00	35.46 ± 3.13	5.69 ± 0.58	-	0.18 ± 0.05	-
	60 V		26.14 ± 2.64	15.61 ± 1.40	11.73 ± 1.74	40.85 ± 0.86	5.39 ± 1.48	-	0.29 ± 0.04	-
C—Na_2_WO_4_	**20 V**		**47.78 ± 7.84**	**6.90 ± 0.40**	**24.35 ± 3.46**	**17.57 ± 11.52**	**3.15 ± 0.48**	**-**	**-**	**0.27 ±> 0.01**
	40 V		30.83 ± 1.26	11.90 ± 1.62	13.03 ± 0.21	37.53 ± 0.42	6.49 ± 0.92	-	-	0.23 ±> 0.01
	60 V		21.84 ± 2.69	16.73 ± 0.82	15.81 ± 0.05	38.72 ± 7.30	6.79 ± 0.01	-	-	0.11 ±> 0.01

**Table 5:** Bandgap (eV) of anodic oxide prepared in reference and modified electrolytes with corresponding wavelength (nm) and region of sunlight.

Electrolyte Name	Voltage (V)	BG (eV)	Corresponding Wavelength (nm)	Range of Sunlight
Reference electrolyte	20 V	3.42	363.03	UV
	40 V	3.14	395.41	UV
	60 V	2.85	435.64	Vis
A—Mn(NO_3_)_2_	20 V	3.30	376.23	UV
	40 V	3.16	392.90	UV
	**60 V**	**2.55**	**486.89**	**Vis**
B—Na_2_MoO_4_	20 V	3.55	349.74	UV
	40 V	3.21	386.78	UV
	60 V	2.92	425.20	Vis
C—Na_2_WO_4_	20 V	3.61	343.93	UV
	40 V	3.15	394.15	UV
	60 V	2.95	420.87	Vis

## Data Availability

The data presented in this study are available on request from the corresponding author. The data are not publicly available due to basic character of research.
